# Patient-initiated transmissions in remote monitoring of cardiac implantable electronic devices: Evaluating volume, clinical value, and short message service-based strategy to reduce unnecessary transmissions

**DOI:** 10.1016/j.hroo.2025.10.011

**Published:** 2025-10-27

**Authors:** Jaakko Huovinen, Jarkko Karvonen, Aapo Aro, Markus Sane

**Affiliations:** Heart and Lung Center, Helsinki University Hospital, and University of Helsinki, Helsinki, Finland

**Keywords:** Pacemaker, Remote monitoring, Transmission, Cardiac implantable electronic devices, Patient-initiated

## Abstract

**Background:**

Remote monitoring (RM) has been shown to improve clinical outcomes compared with traditional in-office follow-up and has seen rapid adoption. However, most RM transmissions are nonactionable, contributing to clinical workload. Many cardiac implantable electronic devices (CIEDs) allow patient-initiated transmissions, which are frequently nonactionable. More efficient strategies are needed to manage this burden without compromising patient safety.

**Objective:**

This study aimed to describe the reasons behind patient-initiated transmissions in RM of CIEDs and assess the impact of a simple short message service (SMS)-based intervention to reduce unnecessary transmissions.

**Methods:**

At Helsinki University Hospital in 2024, each RM transmission was evaluated for its cause and resulting clinical action. Patient-initiated transmissions were analyzed for indications and outcomes. Beginning in June 2024, patients who sent transmissions without a clear medical reason received an SMS explaining RM procedures and device functionality. The effect of this intervention on transmission volume was measured.

**Results:**

4020 CIEDs produced 8182 RM transmissions. Of the 8182 transmissions, 2268 (27.7%) were patient initiated, with an average of 0.049 transmissions per device per month. Among 1234 transmissions triggered by symptoms or unknown reasons, 85.0% were clinically nonactionable. Only 3.6% led to an in-office visit. After implementing the SMS intervention, the frequency of asymptomatic patient-initiated transmissions decreased by 31.2% (*P* = .008).

**Conclusion:**

Patient-initiated transmissions significantly contribute to RM workload, although most do not lead to clinical action. A simple SMS-based strategy effectively reduced nonactionable transmissions, enhancing the efficiency of RM workflows.

**Key Findings**

▪ Patient-initiated transmissions accounted for 27.7% of all remote monitoring (RM) transmissions, but the majority (85%) were clinically nonactionable.
▪ Only 3.6% of symptom- or unknown reason–triggered transmissions resulted in an in-office visit.
▪ Implementation of a simple short message service (SMS) intervention reduced asymptomatic patient-initiated transmissions by 31.2% (*P* = .008).
▪ The SMS-based strategy improved RM efficiency without compromising patient safety.

## Introduction

Remote monitoring (RM) capabilities are commonly found in contemporary cardiac implantable electronic devices (CIEDs). Increasing evidence supports the clinical, economic, environmental, and safety benefits of RM over conventional in-office monitoring.^1^, ^2^, ^3^, ^4^, ^5^, ^6^ In particular, RM has been shown to improve survival rates in patients with high-energy devices, particularly in terms of all-cause mortality and the composite endpoint of mortality and heart failure hospitalizations.^4^^,^^7^^,^^8^ Furthermore, patient adherence to continuous RM significantly correlates with improved survival.^9^ As a result, RM has become a standard of care for CIED patients.^10^ This has led to a growing number of CIEDs in RM and an increasing volume of RM transmissions. The large volume of transmissions has emerged as one of the primary challenges faced by RM clinics.^11^ The workload associated with RM, especially in high-volume centers, is substantial,^12^^,^^13^ highlighting the need for more efficient and safe RM follow-up protocols.

RM transmissions can be either scheduled or nonscheduled. Nonscheduled transmissions are initiated by the patient either when symptoms or clinical events occur (patient-initiated transmissions) or when certain programmed parameters trigger an alert (alert-initiated transmissions). Patient-initiated transmissions can account for up to 22% of the total RM transmissions in a clinic, and most of these transmissions are nonactionable.^13^^,^^14^ In a recent survey study, managing patient phone calls, reviewing remote transmissions, and patient education were among the most substantial perceived burdens for staff.^15^ Therefore, efforts to reduce the frequency of these transmissions should be made.

A known factor to increase asymptomatic patient-initiated transmissions is a lack of device feedback.^16^ Other factors, such as younger age (<75 years), recent device implantation (<1 month), and smartphone-based connectivity, have also been recognized to increase these transmissions.^17^ However, there are limited data on how to effectively reduce the frequency of nonactionable patient-initiated transmissions.

This study aimed to investigate the underlying causes of patient-initiated transmissions and analyze the actions triggered by these transmissions. In addition, the study introduces a simple approach to reduce nonactionable patient-initiated RM transmissions. The findings from this study may offer valuable strategies to help alleviate the RM interpretation burden.

## Methods

### Patient population

Data collection occurred over a 12-month period, from January to December 2024. For the short message service (SMS) analysis, data were collected from January to May 2024 and compared with data collected from January to May 2025.

The study population consisted of CIED patients followed by the Heart and Lung Center of Helsinki University Hospital. This tertiary center is responsible for monitoring all implantable cardioverter-defibrillators (ICDs), cardiac resynchronization therapy (CRT) devices, and implantable loop recorders (ILRs) in the Helsinki and Uusimaa hospital district, which serves approximately 1.8 million inhabitants. In addition, pacemaker (PM) patients from Helsinki, with a population of approximately 680,000, are also monitored at this center.

RM of ICD and CRT devices began at the Helsinki and Uusimaa Heart and Lung Center in 2008, whereas PM patients have been primarily followed by RM since 2020.

At this CIED clinic, all patients are routinely followed up by annual scheduled RM transmissions. Only patients who are unsuitable for RM owing to other comorbidities, refuse RM, or do not have a functioning automatic ventricular pacing threshold management algorithm are followed up in-office. Details of the study population are presented in Table 1.

**Table 1:** Details of patient population during 2024 follow-up∗

Device type	Number of device	Mean age (y)	Transmissions	Sex, male/female (%)
PM	1745	75	368 alert	50.3/49.7
			1798 scheduled	
			628 patient initiated	
ICD	1253	59	764 alert	70.1/29.9
			1091 scheduled	
			1097 patient initiated	
CRT-P	434	77	165 alert	66.7/33.3
			441 scheduled	
			148 patient initiated	
CRT-D	650	64	591 alert	65.3/31.7
			696 scheduled	
			395 patient initiated	

CRT-D = cardiac resynchronization therapy device – defibrillator; CRT-P = cardiac resynchronization therapy device – pacemaker; ICD = implantable cardioverter-defibrillator; PM = pacemaker.

∗ This table contains cumulative number of devices during the follow-up period: 4082 cardiac implantable electronic devices.

### Data collection

In this RM clinic, trained nurses conducted the primary analysis and interpretation of RM transmissions. The cause of each transmission was systemically classified and stored using a standardized data sheet. Transmissions were categorized based on their type (alert, scheduled, or patient initiated), manufacturer, and device type (ICD, CRT – PM, CRT – defibrillator, PM, or ILR). In addition, the reasons for each transmission and the corresponding actions were recorded.

This study focused on analyzing patient-initiated transmissions from ICD, CRT, and PM devices. Biotronik CIEDs were excluded from this analysis because they do not support patient-initiated transmissions. However, Biotronik devices were included in the calculation of the total RM workload.

ILRs were also excluded because their primary indication is symptom correlation, making patient-initiated transmissions essential in their monitoring.

**Figure 1:**
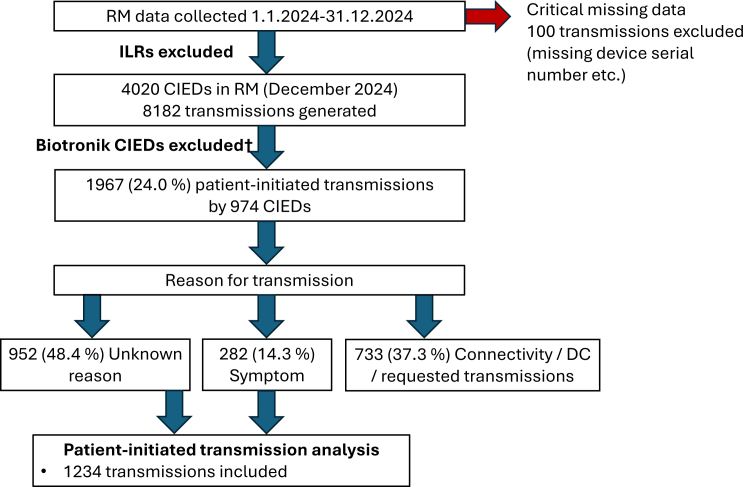
Pathway into patient-initiated transmission analysis. †Biotronik CIEDs do not support patient-initiated transmission. In the data collection setup, transmissions from Biotronik devices that occurred upon the initiation of RM were classified as patient-initiated transmissions, even though they were not triggered by genuine patient actions. CIED = cardiac implantable electronic device; DC = device check; ILR = implantable loop recorder; RM = remote monitoring.

This study included patient-initiated transmissions triggered by a symptom or an unknown reason. Patient-initiated transmissions generated as part of a follow-up protocol—such as connectivity transmissions, device check transmissions, and transmissions requested by the PM clinic—were excluded (Figure 1).

The workload of RM transmissions is reported as transmissions per device per month (/D/Mo).

This value was calculated by dividing the total number of transmissions by the mean number of all devices (including Biotronik) during the follow-up period and the number of follow-up months.

**Figure 2:**
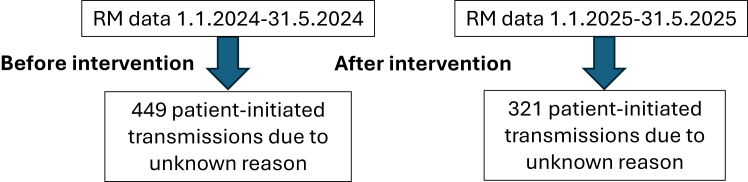
Analysis of short message service intervention effect on patient-initiated transmissions. RM = remote monitoring.

For the SMS analysis, the RM transmission frequency was calculated by excluding Biotronik and ILR devices from the total number of CIEDs. Only patient-initiated transmissions classified as “unknown reason” were included. The number of CIEDs under RM was determined on a month-by-month basis. The effectiveness of the intervention was assessed by comparing transmission rates before and after its implementation. Data were collected from January to May 2024 and January to May 2025 (Figure 2).

Triggered actions after transmissions were classified as nonactionable if no changes in patient care were made, and no patient contact occurred. In our practice, patients are instructed to contact RM staff if any symptoms attributable to the CIED have emerged. If, based on the contact, the symptoms do not seem to be device related, a remote transmission is not requested. If a patient did not contact the PM clinic before initiating a transmission owing to symptoms, the transmission was classified as “unknown reason.”

RM data were obtained from web-based communication systems provided by device manufacturers (Abbott Merlin.net, Medtronic CareLink, Boston LATITUDE).

Clinical information relevant to this study was retrieved from the electronic health record system Apotti (Epic Systems Corporation).

### SMS intervention

Since June 2024, every patient who initiated a transmission owing to an unknown reason has received an SMS providing information about the RM follow-up protocol and device function:

“You have initiated a transmission without a known reason. Your pacemaker is functioning correctly. We would like to remind you that its function is continuously monitored through scheduled transmissions, and if any significant changes occur, the device will automatically send an alert. Therefore, self-initiated transmissions are not necessary as a precaution unless you experience new or clear symptoms. In such cases, we recommend contacting the pacemaker clinic first.”

This intervention was performed by trained nurses after receiving a patient-initiated transmission of unknown reason.

One objective of this study was to evaluate the effectiveness of this intervention in reducing asymptomatic patient-initiated transmissions owing to an unknown reason. Data for the “before intervention” period were collected from January to May 2024, and data for the “after intervention” period were collected from January to May 2025.

We assumed that, after the implementation of the intervention, patients would be more likely to contact the PM clinic before performing a patient-initiated transmission. This behavioral change was expected to result in an increased rate of symptom-triggered transmissions. Data for this outcome were collected over the same periods: January to May 2024 (“before intervention”) and January to May 2025 (“after intervention”).

### Statistics

Statistical analyses were performed using IBM SPSS 29.0.1.0 for Windows (IBM Corp, Armonk, NY). Data visualizations were created using 32-bit Microsoft Excel for Microsoft 365 (version 2502, Build 16.0.18526.20144), IBM SPSS 29.0.1.0 for Windows, Microsoft PowerPoint for Microsoft 365 (version 2505, Build 18827.20164), and Microsoft Word for Microsoft 365 (version 2505, Build 18827.20164).

For the SMS analysis, it was hypothesized that the patient-initiated transmission rate would decline after the introduction of the SMS intervention. The independent variable was the initiation of the SMS intervention, whereas the dependent variable was the patient-initiated transmission rate (/D/Month).

Owing to the intervention's reactive nature, the dependent variable was assumed to follow a non-normal (asymmetrical) distribution. Therefore, the Mann-Whitney U test was chosen for statistical analysis.

## Results

### Transmissions

As of January 2024, a total of 3725 CIEDs were under RM. By the end of the study period in December 2024, the number of CIEDs had increased by 7.9% (4020). During the study period, a total of 8182 transmissions were generated. The frequency of transmissions by device type and indication is presented in Table 2.

**Table 2:** Frequency of different alerts by device type

Type of transmission	Number (%)	Frequency (/D/Mo)				
		All	ICD	CRT-P	CRT-D	PM
Alert	1888 (23.1)	0.041	0.016	0.004	0.013	0.008
Scheduled	4026 (49.2)	0.087	0.024	0.010	0.015	0.039
Patient initiated∗	2268 (27.7)	0.049	0.024	0.003	0.009	0.014
Total	8182 (100.0)	0.176	0.064	0.016	0.036	0.060

/D/Mo = per device per month; CRT-D = cardiac resynchronization therapy device – defibrillator; CRT-P = cardiac resynchronization therapy device – pacemaker; ICD = implantable cardioverter-defibrillator; PM = pacemaker; RM = remote monitoring.

∗ Biotronik devices were included in the calculation of the total RM workload. In the data collection setup, transmissions from Biotronik devices that occurred upon the initiation of RM were classified as patient-initiated transmissions, even though they were not triggered by genuine patient actions. These transmissions are included in this table.

Notably, patient-initiated transmissions accounted for a significant workload, comprising 27.7% of all transmissions.

ICD patients were more likely to initiate transmissions than PM patients. In particular, 58.7% of patient-initiated transmissions in ICD patients were for an unknown reason compared with 48.4% in the overall population.

### Actions after patient-initiated transmissions

During the study period, 2268 patient-initiated transmissions were recorded. After excluding Biotronik transmissions and those that were part of routine follow-up protocols—such as device check transmissions, connectivity tests, and transmissions requested by the PM clinic—1234 transmissions remained. The remaining transmissions were triggered either by a symptom or for an unknown reason. These originated from 584 CIEDs, representing 14.5% of the clinic’s remotely monitored devices.

Biotronik devices were excluded because they do not support patient-initiated transmission (Figure 1).

Approximately 85% of patient-initiated transmissions owing to a symptom or an unknown reason were nonactionable. When considering only transmissions initiated for unknown reasons, the proportion of nonactionable transmissions rose to 94.5%.

The most common action after a patient-initiated transmission was a telephone call to the patient (7.6%) (Table 3).

**Table 3:** Actions after patient-initiated transmissions

Action after patient-initiated transmission	Unknown reason or symptom (%)	Reason for transmission	
		Unknown reason (%)	Symptom (%)
Nonactionable	1046 (84.8)	900 (94.5)	146 (51.2)
In-office follow-up	44 (3.6)	12 (1.3)	32 (11.3)
Telephone call to a patient	94 (7.6)	25 (2.6)	69 (24.5)
Change in drug therapy	13 (1.1)	4 (0.4)	9 (3.2)
Scheduled transmission	14 (1.1)	4 (0.4)	10 (3.5)
Multiple actions∗	16 (1.3)	3 (0.3)	13 (4.6)
Missing data	7 (0.6)	4 (0.4)	3 (1.1)
Total	1234 (100.0)	952 (100.0)	282 (100.0)
Number of individual devices†	584	436	200

∗ “Multiple actions” refers to several interventions resulting from a single transmission—for example, contacting the patient, performing ablation therapy, or programming cardioversion.

† 52 cardiac implantable electronic devices generated both asymptomatic and symptom-triggered patient-initiated transmissions.

### In-office visits after patient-initiated transmissions

During the study period, 1234 patient-initiated transmissions were generated owing to a symptom or an unknown reason. Of these transmissions, 44 (3.6%) resulted in an in-office visit. We analyzed the reasons for requiring an in-office visit and the corresponding actions taken (Table 4).

**Table 4:** The reasons for requiring an in-office visit after a patient-initiated transmission and the following actions performed

Reason for transmission	Transmissions	Actions performed	Transmissions
No symptom/not related to CIED	14	Change in CIED settings	5
		No action	7
		Change in ICD discriminators	1
		Generator change	1
Atrial arrhythmia	9	Cardioversion	4
		Change in CIED settings	3
		Change in medication	1
		No action	1
Ventricular arrhythmia	9	Change in CIED settings	2
		Cardioversion	2
		Change in ICD discriminators	2
		No action	2
		Change in medication	1
Bradycardic symptom	7	Change in CIED settings	7
Device vibration or sound	3	Generator change	2
		No action	1
Heart failure decompensation	1	Change in CIED settings	1
Inadequate ICD therapy	1	Change in medication	1

CIED = cardiac implantable electronic device; ICD = implantable cardioverter-defibrillator.

According to our assessment, the issue triggering the need for an in-office visit would not have come to light without the patient-initiated transmission in 4 cases presented in Table 5.

**Table 5:** Clinically significant problems undetected by a CIED but revealed through patient-initiated transmissions

Case	Clinical background
Case 1	A patient with a DDD-ICD implanted for dilated cardiomyopathy developed repetitive pacemaker-mediated tachycardia. This issue was initially managed by changing the bradycardia pacing mode to AAI-ICD. However, the patient later developed a clinically significant atrioventricular conduction disorder and experienced bradycardic symptoms. The patient initiated a transmission because of these symptoms, which led to the detection of the conduction disorder. The problem was resolved by reprogramming the cardiac device settings.
Case 2	A patient with an ICD for hypertrophic cardiomyopathy experienced persistent slow ventricular tachycardia that the device failed to detect. The patient performed a patient-initiated transmission, which revealed the ongoing ventricular tachycardia on the intracardiac electrogram. The issue was resolved by adjusting the detection settings of the device and optimizing antiarrhythmic medications.
Case 3	A patient with a DDD pacemaker implanted for atrioventricular conduction disorder developed a pacemaker Wenckebach phenomenon during episodes of sinus tachycardia. This occurred because the postventricular atrial refractory period had been set excessively long owing to previous episodes of pacemaker-mediated tachycardia. The issue was identified through a patient-initiated transmission and was resolved by modifying the device settings.
Case 4	A patient with a DDD pacemaker for atrioventricular conduction disorder developed an atrial flutter that the device failed to recognize. The arrhythmia was undetected owing to an excessively long postventricular atrial blanking period, which resulted in an undersensing of atrial activity. The patient initiated a transmission, leading to the identification of the problem, which was then corrected by reprogramming the device settings.

AAI = atrial chamber pacing system; DDD = dual-chamber pacing system; ICD = implantable cardioverter-defibrillator.

### Most frequent patient-initiated transmission performers

There were 11 patients who generated an especially high number of patient-initiated transmissions—10 or more—during the study period. These patients accounted for a total of 168 transmissions, of which 164 (97.6%) were either asymptomatic or related to noncardiac symptoms. Only 4 transmissions were initiated owing to cardiac symptoms, specifically atrial arrhythmias. The average age of these patients was 43.3 years, which is significantly younger than the overall study population, whose average age was 69.3 years. The mean duration since the initiation of RM among these patients was 46.3 months. Notably, 7 of these 11 patients had Boston Scientific’s subcutaneous ICD (S-ICD), despite S-ICDs comprising only 63 of the total 4020 CIEDs (1.6%) in the entire study cohort.

### SMS intervention

**Figure 3:**
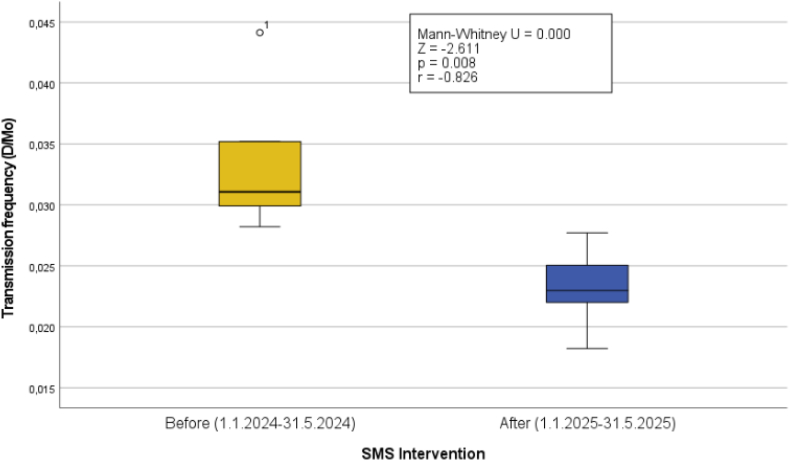
Mann-Whitney U test results comparing patient-initiated transmission frequencies before and after the SMS intervention. SMS = short message service.

The SMS intervention had a significant impact on the transmission frequency. A statistically significant difference was observed between the transmission frequencies before and after the introduction of the SMS intervention (Mann-Whitney U = 0.0; Z = −2.6; *P* = .008; r = −0.8). Implementing the SMS intervention and its effect on transmission frequency is visualized below (Figure 3).

On average, the asymptomatic patient-initiated transmission frequency was 0.03 transmissions/D/Mo before the intervention.

After the SMS intervention was implemented, the asymptomatic transmission frequency decreased by 31.2%, dropping to 0.02 transmissions/D/Mo.

During the same period, symptom-triggered transmissions decreased by 30.3%, dropping from 0.01 to 0.007 transmissions/D/Mo (Mann-Whitney U test *P* = .008).

After the SMS intervention was initiated, 6.3% of asymptomatic transmissions were actionable (before the intervention 5.5%) (Table 3).

The relative reduction of the SMS intervention in transmission rate was similar for frequent transmitters (≥3 transmissions) and infrequent transmitters (<3 transmissions) during the preintervention period, at 77.6% and 77.8%, respectively.

## Discussion

This study provides novel insights into the reasons behind patient-initiated transmissions. These transmissions represented 27.7% of all recorded transmissions (2268). Of these, 1234 transmissions (54.4%) were initiated either because of symptoms or for an unknown reason. Most transmissions (85.0%) were nonactionable, and when considering only those initiated for an unknown reason, the proportion of nonactionable transmissions rose to 94.5%. In-office visits were rarely required (3.6%), and in most cases, issues were resolved by adjusting CIED settings. Given that most patient-initiated transmissions are clinically nonactionable, efforts should be made to reduce their frequency to optimize clinical workflow and resource utilization.

In response, we implemented a simple SMS-based educational interface for patients who initiated transmissions without a clear clinical indication. A lack of device feedback has previously been identified as a factor increasing patient-initiated transmissions.^16^ The SMS intervention specifically targets this issue, reassuring patients about their device function. This strategy led to a significant 31.2% reduction in such transmissions, demonstrating a promising approach to improving the efficiency of RM systems. Among individuals who had submitted transmissions during the preintervention period, the decrease was 77.6% and 77.8% for frequent and infrequent transmitters, respectively. This suggests that, after the intervention, most transmissions were generated by new patients who had not yet been exposed to the SMS intervention. Therefore, an even greater reduction in transmission rates can be anticipated over the longer term.

We anticipated that implementation of the new protocol would require time for patients to learn and adapt. Therefore, we evaluated its effectiveness by comparing the rate of patient-initiated transmissions over a 5-month period in 2024 with the same 5-month period in 2025 to assess the year-over-year change.

We expected an increase in patient-initiated transmissions triggered by symptoms after implementing the SMS intervention, assuming that patients would be more likely to contact the PM clinic before performing a transmission. Surprisingly, even these transmissions decreased by 30.3% after the SMS intervention was introduced. This may be partly explained by the fact that 10% of patients with symptom-triggered transmissions also had asymptomatic transmissions and therefore received the intervention. However, it is important to note that the overall rate of symptom-triggered transmissions was very low (from 0.01 to 0.007 transmissions/D/Mo), and most patients likely do not contact the PM clinic before performing a transmission, even when symptomatic.

When interpreting these results, it is important to note that, in this clinic, atrial tachycardia alerts have been disabled for patients with a known history of atrial fibrillation and ongoing anticoagulation therapy. This may affect the results that, instead of alert transmissions, patients perform symptom-triggered transmissions. The rationale behind this approach is that symptomatic patients with atrial fibrillation typically seek cardioversion on their own, without an alert-initiated transmission, and rarely perform patient-initiated transmissions. Unfortunately, we did not collect data on how many patient-initiated transmissions were related to atrial fibrillation.

A noteworthy finding in this study is the overrepresentation of patient-initiated transmissions in Boston Scientific’s S-ICD devices. This is likely caused by a technical issue that makes it easy to accidentally initiate a transmission. Previous studies have also reported a high proportion of patient-initiated transmissions with S-ICDs.^18^ Improving this technical limitation in future device versions could substantially reduce the burden of interpretation.

There are clear benefits to having the patient-initiated transmission function in CIEDs. In some cases, these transmissions reveal critical clinical issues that would otherwise go undetected. In addition, they provide valuable information on symptom correlation with device function or intracardiac electrogram. Symptom-triggered recordings with symptom correlation can save costs and enhance patient safety by reducing the need for potentially harmful invasive procedures.

In the future, more flexible communication channels for CIED patients in RM could improve patient reassurance and satisfaction. One potential approach is the introduction of a secure, bilateral communication channel via a smartphone-based RM application. This would allow patients to receive direct feedback about their symptoms and offer health care professionals a more convenient and efficient means of communication.

### Strengths and weaknesses

This study was conducted at a tertiary care hospital with extensive experience in managing a diverse population of CIED patients. The standardized RM follow-up protocol ensured high-quality data collection, providing a strong foundation for analysis. The study is based on real-world clinical practice, increasing its relevance to daily RM workflows. The SMS intervention is an innovative and low-cost strategy to reduce unnecessary transmissions, offering practical implications for other RM clinics. However, certain limitations in data collection, study design, and analysis are acknowledged.

Given that data collection was performed manually, there is a risk of errors and gaps in the dataset. Altogether, 100 critical data absences led to the exclusion of those transmissions from the analysis. These absences included missing information on device number, manufacturer, device type, transmission type, or primary reason for transmission. However, given that these omissions accounted for only 1.2% of all transmissions during the study period, the overall data quality remains high.

This study also has statistical limitations. In the SMS intervention analysis, time was used as a surrogate variable to assess the impact of the intervention on patient-initiated transmissions. However, this approach does not account for potential confounding factors that may have influenced transmission rates. This intervention study did not involve randomization nor a control group and is therefore vulnerable to causal interference. It should also be noted that the study does not explore whether the SMS intervention affected patient anxiety, satisfaction, or trust in RM, which could be important for long-term adherence. Although the intervention showed a significant reduction in transmission frequency, long-term effects on patient behavior and RM workload remain unknown.

## Conclusion

Patient-initiated transmissions contribute significantly to the interpretation burden in RM but rarely lead to clinically significant findings. Implementing an SMS-based intervention can effectively reduce the number of nonactionable transmissions.

## Disclosures

J.H.: grant by the Finnish Foundation for Cardiovascular Research. J.K.: speaker honoraria and/or consultancy fees from Abbott, Biotronik, Boston Scientific, and Medtronic and advisory board membership at Medtronic. A.A.: grant by the Finnish Foundation for Cardiovascular Research. M.S.: speaker honoraria and/or consultancy fees from Abbott, Biotronik, and Medtronic and travel grants from Biotronik. During the preparation of this work, the authors used *OpenAI ChatGPT* to improve readability and language. After using this tool, the authors reviewed and edited the content as needed and take full responsibility for the content of the publication.
